# Protocol for identifying surface membrane proteins and their associated proteome from mouse cortical neuron cultures by *in situ* biotinylation

**DOI:** 10.1016/j.xpro.2026.104418

**Published:** 2026-03-09

**Authors:** Liang Shi, Pritha Bagchi, GiaLinh N. Nguyen, Victor Faundez, Gary J. Bassell

**Affiliations:** 1 Department of Cell Biology, Emory University School of Medicine, Atlanta, GA 30322, USA; 2 Emory Integrated Proteomics Core, Atlanta, GA 30322, USA

**Keywords:** Cell membrane, Cell separation/fractionation, Mass spectrometry

## Abstract

Modifications to the plasma membrane proteome reflect neuronal physiological states. Here, we present a protocol to profile surface proteins from mouse cortical neuron cultures using *in situ* biotinylation. The workflow includes neuron culture, surface labeling, enrichment of biotinylated proteins, and on-beads digestion followed by mass spectrometry, enabling quantitative and comparative analysis of membrane-associated proteins across diverse physiological conditions.

For complete details on the use and execution of this protocol, please refer to Shi et al.^1^

## Highlights

• Instructions for labeling membrane proteins *in situ* in live neurons
• Procedures for purifying membrane and membrane-associated proteins
• Steps for identifying membrane proteins that report local membrane events

## Abstract (editor highlights)

Publisher’s note: Undertaking any experimental protocol requires adherence to local institutional guidelines for laboratory safety and ethics.

## Graphical abstract

**Figure:**
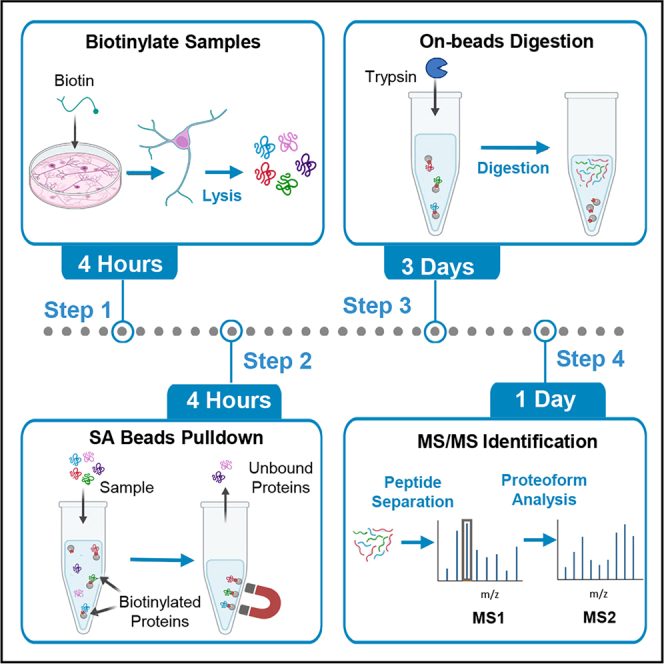

## Teaser

Modifications to the plasma membrane proteome reflect neuronal physiological states. Here, we present a protocol to profile surface proteins from mouse cortical neuron cultures using *in situ* biotinylation. The workflow includes neuron culture, surface labeling, enrichment of biotinylated proteins, and on-bead digestion followed by mass spectrometry, enabling quantitative and comparative analysis of membrane-associated proteins across diverse physiological conditions.

## Before you begin

The quality of neuronal cultures is extremely important for membrane fractionation, affecting both yield and purity. Dead cell debris can contaminate the final membrane protein samples with cytosolic proteins or proteins from organelles other than the plasma membrane, while immature neurons can significantly alter membrane protein distribution. To ensure high quality cultures, supporting glial cells are prepared four weeks before embryonic dissection, and poly-L-lysine coated dishes are prepared two days before neuronal plating. Once the glial conditioned medium and coated dishes are ready, E17 mouse embryos are carefully dissected, and cortical neurons are cultured in glial conditioned medium for 14–21 days to obtain mature, healthy neurons. If needed, neuronal maturity can be verified by immunofluorescence staining using markers such as PSD-95 or Synapsin-1.

### Innovation

For decades, membrane protein purification has traditionally relied on cell fractionation by differential and equilibrium sedimentation. With the development of live-cell biotinylation using reactive biotin reagents specific for different chemical groups present in proteins and glycans, surface membrane proteins can now be efficiently labeled, and membrane-associated proteins can subsequently be co-isolated using streptavidin-coated magnetic beads. Unlike most *in situ* labeling methods, such as BioID, which focus on mapping the interactome of a specific protein of interest, this approach enables the preparation of comprehensive membrane-associated protein samples from neuronal cultures, where much of the membrane fraction originates from neurites. The following protocol details the specific steps for use with primary cortical neuron cultures, but it can also be adapted for other cell types or tissue preparations.

### Institutional permissions

All animal studies were carried out in compliance with protocols approved by the Emory University Institutional Animal Care and Use Committee (IACUC) and Biosafety Committee. Experiments involving animal tissues were conducted in designated BSL-2 facilities at Emory under the required institutional authorizations. Before initiating any animal-related procedures outlined in this protocol, researchers should obtain the necessary ethical and institutional approvals.

### Preparation of glial conditioned medium (GCM+)

**Timing: 4 weeks**

Primary neurons grow and mature more effectively with support from astrocytes. While direct neuron-glia co-culture can be used in some experiments, it is not suitable for our purposes, as glial cells would contaminate the neuronal population. Instead, astrocytes are isolated from embryonic mouse brains, expanded in culture, and used to precondition the neuronal culture medium. Neurobasal+ medium supplemented with B27+ is incubated with glia, allowing it to accumulate glia-secreted factors. Using this glial conditioned medium, healthy and mature cortical neurons are obtained within two weeks.

1. Dissection.
  a. Sacrifice E17 mouse according to IACUC approved procedure.
  b. Remove all embryos from E17 mouse and place them into a sterile Petri dish containing ice-cold HBSS/HEPES (Corning 21022CV, Cytiva SH30237.01).
  c. Transfer only the heads to another sterile Petri dish containing ice-cold HBSS/HEPES.
  d. Perform brain dissection and isolate cortical tissues from embryonic brains.
  e. Digest the tissue by placing it into a 15 mL conical tube containing 2.5 mL of0.25% Trypsin (Thermo Scientific, 15050–065) (prewarmed to 37°C) for 10 min.
  f. Transfer the tissue from the trypsin to a 15 mL conical tube containing 5 mL of HBSS/HEPES (prewarmed to 37°C) to rinse; repeat 1 more time.
  g. Place tissues in 5 mL of incubated MEM/FBS *Attachment* medium (Corning 10010CV, Gibco A3160402) and triturate 20–25 times with a sterile 6 mL plastic transfer pipette until a single-cell suspension is formed.
  h. Filter the single-cell suspension through a 100 μm cell strainer.
  i. Count the number of live neurons by Trypan Blue staining (Invitrogen, T10282).
2. Growing primary glia.
  a. Combine the single-cell suspension with 10 mL of MEM/FBS medium in a T75 flask.
  b. Incubate in a cell culture incubator (37°C, 5% CO2, 95% relative humidity) for 24 h.
  c. Completely remove the old MEM/FBS medium and add fresh pre-incubated MEM/FBS medium.
  d. Replace the medium every 7 days until the glial culture reaches 100% confluence.
3. Preparing secondary glia.
  a. Remove the MEM/FBS medium from the 100% confluent glia flask and then wash 2 times with 10 mL HBSS/HEPES (prewarmed to 37°C).
  b. Add 5 mL 0.25% trypsin with EDTA to the flask and place into a cell culture incubator (37°C, 5% CO2, 95% relative humidity) for 5 min.
  c. Tap flask and check under a microscope to ensure cells are suspended.
  d. Add 20 mL of MEM/FBS medium to the flask to stop the trypsin reaction and triturate.
  e. Pipette the mixture into a 50 mL conical and centrifuge cells at 1,000 x *g* for 5 min.
  f. Remove the supernatant and resuspend the cell pellet with 12.5 mL MEM/FBS.
  g. Add 500 μl of the cell suspension to each well of 6-well plates.
  h. Replace the MEM/FBS medium every 7 days until the culture reaches 70–90% confluence.

**CRITICAL:** Do not use 100% confluent glia, since the glia will consume all available glucose in the feeding medium.

4. Collecting glial conditioned medium (GCM+).
  a. Remove the MEM/FBS medium & replace with Neurobasal+/B27+/Glutamax *Feeding* medium (Gibco A3582901, Gibco A3582801, Gibco 35050061).
    ***Note:*** GCM+ is a feeding medium that has been conditioned by secondary glia. B27+ (1:50) should be freshly added to Neurobasal+/GlutaMAX (500 mL + 5 mL) mixture when ready to feed secondary glia.
  b. Culture the secondary glia in the feeding medium for 7 days.
    **CRITICAL:** At least one medium change must be performed before collecting the GCM+ for the first time.
  c. Replace the feeding medium the day before collecting the GCM+ and allow it to incubate for 16–20 h.
  d. Collect the GCM+ and immediately use it to feed cells. Alternatively, it can be collected and stored at −20°C for up to 12 months.
    ***Note:*** You can collect GCM+ for 6–8 weeks before the secondary glia cultures need to be discarded.

### Preparation of poly-L-lysine-coated dishes

**Timing: 2 days**

Neurons are highly sensitive to the culture surface, and suboptimal coating can lead to unhealthy cultures that have clumped, detached, or degenerating cells. Proper coating with poly-L-lysine ensures an optimal substrate, supporting the attachment, growth, and maturation of primary mouse cortical neurons more efficiently and reliably.

5. Dilute 1 mg/mL poly-L-lysine (PLL, Sigma P2636) 50x stock in Borate Buffer (pH 8.5) to make 20 μg/mL PLL 1x working solution.
6. Add 5 mL of PLL 1x working solution into one 10 cm dish and incubate in a cell culture incubator for 16–20 h.
7. Wash the 10 cm dish with 10 mL of sterile water for 1 h on an orbital shaker; repeat 2 more times.
8. Add 10 mL of MEM/FBS medium and incubate in a cell culture incubator for 16–20 h.

**CRITICAL:** A cell culture incubator set to 37°C, 5% CO2, 95% relative humidity must be used to incubate medium used directly with neuronal cultures. Incubation allows the medium to reach ideal temperature, aeration, and pH level (37°C, 5% CO2, pH 7.4) to grow neurons. Using unincubated medium stresses neurons and results in poor adhesion and growth. **Troubleshooting 1**.

***Note:*** Coated culture dishes can be kept in a cell culture incubator for up to 1 week.

***Optional:*** Considering the different cell types, other coating reagents can be used accordingly, such as Matrigel or laminin.

### Culturing primary cortical neurons

**Timing: 2 weeks**

Rapid dissection and the use of optimized glial conditioned medium (GCM+) are critical for establishing healthy primary neuron cultures and achieving high yields of neuronal proteins for downstream analyses.

9. Plate 2 million cortical neurons from the single-cell suspension in one 10 cm PLL coated dish and incubate for 2 h in MEM/FBS medium.
10. Replace MEM/FBS medium with 10 mL of prewarmed incubated GCM+.

**CRITICAL:** Glial conditioned medium (GCM+) is used in this protocol. To achieve healthy neuron cultures, GCM+ is highly recommended. The advantages and preparation of glial conditioned medium can be seen in previous study by Zheng et al.^2^

***Optional:*** Other neuronal culture media can be used if necessary.

11. Feed neurons twice a week and culture for at least 14 days in GCM+ before proceeding to the biotinylation procedure.

**CRITICAL:** Do not proceed to the next step if neurons do not look healthy, are not proliferating as expected, or if other cell contamination is present. **Troubleshooting 2**.

## Key resources table

**Table:** 

REAGENT or RESOURCE	SOURCE	IDENTIFIER
**Chemicals, peptides, and recombinant proteins**		

EZ-Link Sulfo-NHS-SS-Biotin	Thermo Scientific	Cat# 21331
Halt Protease and Phosphatase Inhibitor Cocktail	Thermo Scientific	Cat# 78441
Dynabeads Protein G beads	Invitrogen	Cat# 10004D
Dynabeads M-280 Streptavidin	Invitrogen	Cat# 11205D
Poly-L-Lysine Hydrobromide (mol wt 30,000–70,000)	Sigma-Aldrich	Cat# P2636
Neurobasal Plus Medium	Gibco	Cat# A3582901
B27 Plus	Gibco	Cat# A3582801
GlutaMAX Supplement (100x)	Gibco	Cat# 35050061
4–20% Mini-PROTEAN TGX Precast Protein Gels	Bio-Rad	Cat# 4561093
Nitrocellulose Membrane, 0.2 μm	Bio-Rad	Cat# 1620112
Normal Donkey Serum	Jackson ImmunoResearch	Cat# 017-000-121, RRID:AB_2337258
ProLong Gold Antifade Mountant with DAPI	Invitrogen	Cat# P36935
MEM	Corning	Cat# 10010CV
Fetal Bovine Serum, US Certified, One Shot - FBS	Gibco	Cat# A3160402
Glucose (>99.5% purity)	Sigma-Aldrich	Cat# G8270
Hank’s Balanced Salt Solutions - HBSS	Corning	Cat# 21022CV
HEPES Buffer Solution 1M	Cytiva	Cat# SH30237.01
Trypsin (0.25%), phenol red	Gibco	Cat# 15050065
Trypsin (0.25%) with EDTA	Gibco	Cat# 25200-056
Trypan Blue	Invitrogen	Cat# T10282
Boric Acid (>99.5%, molecular biology grade)	Sigma-Aldrich	Cat# B6768
Sodium Tetraborate Decahydrate (>99.5% purity)	Sigma-Aldrich	Cat# S9640
10X PBS	Thermo Scientific	Cat# J75889.K8
Glycine (>99% purity)	Sigma-Aldrich	Cat# 50046
Ammonium Bicarbonate (>99.5% purity)	Sigma-Aldrich	Cat# 09830
Dithiothreitol - DTT (>99%, molecular biology grade)	Sigma-Aldrich	Cat# D5545
Iodoacetamide (molecular grade)	Sigma-Aldrich	Cat# I1149
Lysyl Endopeptidase (mass spectrometry-grade)	Fujifilm/Wako	Cat# 125-05061
Trypsin Protease (mass spectrometry-grade)	Thermo Scientific	Cat# 90059
Oasis HLB 1 cc Vac Cartridge Column	Waters	Cat# WAT094225
Trifluoroacetic Acid	Sigma-Aldrich	Cat# T6508
Formic Acid, LC-MS Grade (mass spectrometry-grade)	Thermo Scientific	Cat# 85178
Acetonitrile, Optima™ LC/MS Grade	Thermo Scientific	Cat# A9554

**Critical commercial assays**		

Pierce ECL Western Blotting Substrate	Thermo Scientific	Cat# 32106
Pierce BCA Protein Assay Kits	Thermo Scientific	Cat# 23227

**Deposited data**		

Affinity AP2 Proteomics Fragile X	ProteomeXChange Consortium	Database: PXD065502

**Experimental models: organisms/strains**		

Experimental mouse: E17 female WT C57BL/6J	Charles River Laboratories	Strain# 027; RRID:IMSR_CRL:027

**Software and algorithms**		

Proteome Discoverer 2.1	Thermo Scientific	RRID:SCR_014477
Metascape	Zhou et al., 2019	RRID:SCR_016620
MCODE	Bader GD, Hogue CW., 2003	RRID:SCR_015828

**Other**		

Orbitrap Fusion Lumos Mass Spectrometer	Thermo Scientific	Cat# FSN04-10001
Dionex UltiMate™ 3000 RSLCnano System	Thermo Scientific	Cat# 5200.0355

## Materials and equipment

HBSS/HEPES (pH 7.3).

• HBSS – 500 mL.
• 1 M HEPES – 5 mL.

Note on storage conditions: Store at 4°C for up to 6 months. Sterile filter before storing.

**Table:** MEM/FBS “*Attachment”* Medium (pH 7.3)

Reagent	Final concentration	Amount
MEM, Corning 10010CV	N/A	500 mL
1 M HEPES	10 mM	5 mL
Glucose	6 g/L	3 g
FBS	10%	50 mL
**Total**	**N/A**	**555 mL**

Note on storage conditions: store at 4°C for up to 6 months. Sterile filter before storing.

**Table:** Neurobasal+/B27+/GlutaMAX “*Feeding”* Medium

Reagent	Final concentration	Amount
Neurobasal+	NA	500 mL
GlutaMAX (100x)	NA	5 mL
B27+	NA	10 mL
**Total**	**N/A**	**515 mL**

Note on storage conditions: Neurobasal+/GlutaMAX can be premixed and store at 4°C for up to 6 months. When ready to make *Feeding* medium, add freshly thawed B27+ (1:50) to the Neurobasal+/GlutaMAX mixture. Co-culture with secondary glia to create Glial Conditioned Medium Plus (GCM+).

**Table:** Borate Buffer (pH 8.5)

Reagent	Final concentration	Amount
Boric acid	40 mM	1.24 g
Sodium Tetraborate	15 mM	2.9 g
ddH2O	N/A	500 mL

Note on storage conditions: store at 4°C for up to 12 months. Sterile filter before storing.

### Poly-L-lysine 1X solution (20 μg/mL)

• Poly-L-lysine 50x stock – 20 μL.
• Borate Buffer (pH 8.5) to make – 1 mL.

Note of storage conditions: Make PLL 1X solution fresh when ready to coat dishes. Sterile 50x stocks of PLL can be stored at −20°C for 2–3 years. Thaw in a 37°C dry bath when ready to use.

**Table:** PBS/Ca/Mg solution

Reagent	Final concentration	Amount
PBS	N/A	100 mL
1M CaCl2	0.1 mM	10 uL
1M MgCl2	1 mM	0.1 mL
**Total**	**N/A**	**100 mL**

Note on storage conditions: store at 4°C for up to 12 months.

### Glycine wash

• 50 mM Glycine – 375 mg of glycine (Sigma 50046).
• PBS/Ca/Mg solution – 100 mL.

Note on storage conditions: store at 4°C for up to 12 months.

**Table:** 10X Buffer A stock (pH 7.4)

Reagent	Final concentration	Amount
5 M NaCl	1 M	30 mL
1 M HEPES	0.1 M	10 mL
1 M EGTA	10 mM	1 mL
1 M MgCl2	1 mM	0.1 mL
ddH2O	N/A	59 mL
**Total**	**N/A**	**100 mL**

Note on storage conditions: Store at 20–22°C for up to 12 months.

### 1X buffer A solution (pH 7.4)

• 10x Buffer A stock.
• ddH2O – add ddH2O to dilute 10x stock solution to 1:10.

Note on storage conditions: Make fresh, final solution of 1X Buffer A solution should be 100 mM NaCl, 10 mM HEPES, 1 mM EGTA, and 0.1 mM MgCl2.

**Table:** Neuron Lysis Buffer

Reagent	Final concentration	Amount
1X Buffer A solution	N/A	500 uL
Triton X-100	0.5%	na
Halt Protease and Phosphatase Inhibitor Cocktail (100x)	N/A	5 uL
**Total**	**N/A**	**500 uL**

Note on storage conditions: Make fresh, multiply by number of samples.

### Buffer A/inhibitor mixture

• 1X Buffer A solution – 500 uL.
• Halt Protease and Phosphatase Inhibitor Cocktail (100x) – 5 uL.

Note on storage conditions: Make fresh.

### Pulldown wash

• 500 μL of 1X Buffer A.
• 0.2% Triton X-100.

Note on storage conditions: Make fresh.

### MS-buffer A

• 0.1% formic acid – 0.1 mL.
• LC-MS grade water – 100 mL.

Note on storage conditions: store at 4°C for up to 12 months.

### MS-buffer B

• 0.1% formic acid – 0.1 mL.
• Acetonitrile – 100 mL.

Note on storage conditions: store at 4°C for up to 12 months.

### Ultra-sonicator settings

• 30% power.
• Ultrasonicate – 3 s.
• Pause – 2 s.
• Repeat 10 times.

***Note:*** Samples should be on ice when sonicating.

## Step-by-step method details

### Preparation of biotinylation samples

**Timing: 4 h**

Biotinylate membrane proteins directly on live neuron cultures. The neurons remain attached to the dish throughout the procedure, avoiding any suspension steps that could damage cells or alter membrane protein localization. Because the cells are not permeabilized, the biotinylation reagent can only access and label proteins exposed on the extracellular surface of the plasma membrane. This is because the biotinylation reagent used is Sulfo-NHS-SS-Biotin. The sulphate group in the reagent renders the molecule cell impermeant. This ensures that only membrane proteins exposed to the extracellular milieu are tagged, with no labeling of intracellular components. As a result, the method provides a selective labeling of surface membrane proteins for downstream pulldown. Note that the precipitation step with streptavidin may pull down intracellular proteins not biotinylated as the product of interactions between a biotinylated membrane protein and a cytoplasmic factor or organelle.

1. Biotinylate neurons.
  a. Rinse neurons twice with ice-cold PBS/Ca/Mg (PBS with 0.1 mM CaCl2 and 1 mM MgCl2). **Troubleshooting 3**.
    **CRITICAL:** Perform all biotinylation procedures with sample on ice.
  b. Incubate neurons with 1 mg/mL EZ-Link Sulfo-NHS-SS-Biotin (Thermo Scientific, 21331) in PBS/Ca/Mg at 4°C for 30 min.
    i. Just before rinsing the neurons, add stock EZ-Link Sulfo-NHS-SS-Biotin into ice-cold PBS/Ca/Mg.
    ii. Slowly add EZ-Link Sulfo-NHS-SS-Biotin solution to the culture dish until all cells are submerged in the solution.
    iii. Incubate neurons on a rocker for 30 min in the fridge at 4°C.
      ***Note:*** There are two choices of this reagent, Sulfo-NHS-SS-Biotin and Sulfo-NHS-Biotin. The advantage of the disulfide bond (S-S) in Sulfo-NHS-SS-Biotin is that it allows for assessing labeled proteins once the disulfide bond is cleaved with DTT and the biotin molecule is stripped off.
  c. Wash cells with Glycine wash at 4°C for 5 min.
    **CRITICAL:** Glycine washing is critical to quench free Sulfo-NHS-SS-Biotin present in the incubating medium. Glycine offers free NH3 groups that will react with the NHS chemistry in Sulfo-NHS-SS-Biotin. This will prevent artifactual biotinylation of intracellular proteins during cell lysis. Additionally, this wash removes residual Sulfo-NHS-SS-Biotin, which will bind to streptavidin (SA) beads, outcompeting biotinylated proteins thus reducing pulldown efficiency.
    ***Note:*** For the negative control, cells are incubated with just PBS/Ca/Mg solution.
  d. Repeat wash 1 more time.
2. Lyse neurons.
  a. Combine 500 μL of 1X Buffer A with 0.5% Triton X-100 and 5 μL of 100X Halt Protease and Phosphatase Inhibitor Cocktail (Thermo Fisher Scientific, 78441) to make a neuron lysis buffer.
  b. Add neuron lysis buffer to the culture dish.
    **CRITICAL:** To get samples with high protein concentration, it is recommended to use no more than 500 μL neuron lysis buffer for a 10 cm dish and no more than 125 μl neuron lysis buffer for one well of a 6-well plate.
  c. Detach the cells from culture dishes using a cell scraper.
  d. Collect the cell lysate in a 1.5 mL microcentrifuge tube and place it on ice.
  e. Ultrasonicate the lysate samples (settings: 30% power, ultrasonicate 3 s, pause 2 s, repeat 10 times).
    **CRITICAL:** Always keep samples on ice to reduce heat denaturation of protein.
  f. Incubate the lysate on ice for at least 30 min.
  g. Centrifuge the lysate at 16,000 x *g* for 30 min at 4°C.
  h. Collect the supernatant in a new tube.
  i. Measure protein concentrations by BCA assay.
    **CRITICAL:** The minimum usable protein concentration is 0.8 mg/mL. If the protein concentration is less than 0.8 mg/mL, then you will need to restart from the culturing primary cortical neurons step. **Troubleshooting 4**.
  j. Aliquot protein samples, use immediately or flash freeze to store at −80°C.
    ***Note:*** For best result, use the protein lysate immediately after collecting.
    **Pause point:** If the protein yield is insufficient (less than 500 μg) but the protein concentration is good (more than 0.8 mg/mL), samples can be stored at −80°C until enough protein is collected to perform the pulldown procedure. Do not freeze-thaw more than once.
    ***Optional:*** The biotinylation efficiency can be examined by either Western blot or immunofluorescence staining using a fluorescent conjugated streptavidin (see Figure 1 for examples). **Troubleshooting 5.**
    **Figure 1:**
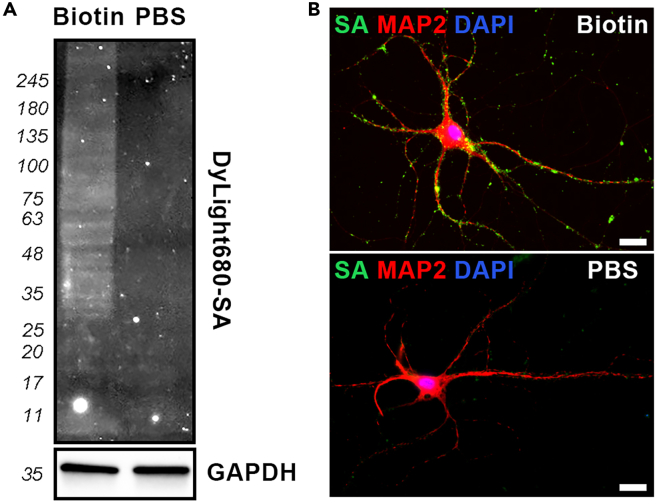
Quality validation of surface protein biotinylation (A) Whole-cell lysates were probed with DyLight 680-streptavidin (SA) to detect biotinylated proteins, with GAPDH used as an internal control. (B) Biotinylated mouse cortical neurons were stained with Alexa Fluor 488-streptavidin (SA) to visualize surface protein labeling, while MAP2 immunostaining shows neuronal morphology. Scale = 20 μm.

### Streptavidin beads pulldown

**Timing: 4 h**

Pull down biotinylated proteins using streptavidin beads. The streptavidin-coated magnetic beads pull down the biotinylated membrane proteins and any associated proteins binding to them through either direct or indirect interactions. Streptavidin-coated Sepharose beads or Agarose beads are not recommended due to higher background.

3. Prepare pulldown samples.
  a. For each sample, add 500 μl of 1X Buffer A with 5 μL of 100X Halt Protease and Phosphatase Inhibitor Cocktail to make the Buffer A/Inhibitor mixture.
  b. Add 500 μg of biotinylated protein sample to a 1.5 mL microcentrifuge tube.
    ***Note:*** If you pool from multiple batches, combine equal amounts from each batch to make 500 μg of biotinylated protein sample. Average the concentration of the batches to estimate the concentration of the sample.
  c. Add enough of Buffer A/Inhibitor mixture to the sample so the final volume of the sample mixture is 1 mL.
  d. Add 10% Triton X-100 into the sample mixture so the final concentration of Triton X-100 is 0.2%. See the protein to media calculation table for an example.
    **CRITICAL:** If the protein concentration is lower than 0.8 mg/ml, you will not be able to dilute Triton X-100 to 0.2% and achieve the 1 mL final sample mixture volume.
    **Table:** Protein to media calculation table

	Formula	Example
Protein Concentration	X	2.5 μg/uL
Protein Amount	500 ug	500 ug
Protein Volume	500/X	200 uL
Buffer A/Inhibitor solution	1000–500/X-(2–2.5/X)	799 uL
10% Triton X-100	2–2.5/X	1 uL
  e. Pre-clear samples.
    i. Add 30 μL of magnetic Protein G beads (Invitrogen, 10004D) to a 1.5 mL microcentrifuge tube for each sample.
    ii. Wash the Protein G beads with 500 μL Pulldown Wash (500 μL of 1X Buffer A, 0.2% Triton X-100) for 5 min.
    iii. Use a magnetic rack to pull the beads to the bottom of the tube and extract the Pulldown Wash. Repeat the wash step 2 more times.
    iv. Add 1 mL of sample to the tube with the pre-washed magnetic Protein G beads and incubate on a rocker for 30 min at 4°C.
      ***Note:*** Pre-clearing samples remove non-specific binding protein by binding it to the agarose beads.
4. Pull down of biotinylated proteins.
  a. Add 50 μL of magnetic Streptavidin (SA) Dynabeads (Invitrogen, 11205D) to a 1.5 mL microcentrifuge tube for each sample.
  b. Wash the SA beads with 500 μL of Pulldown Wash for 5 min.
  c. Use a magnetic rack to pull the beads to the bottom of the tube and extract the Pulldown Wash. Repeat the wash step 2 more times.
  d. Add 1 mL of pre-cleared samples to pre-washed magnetic SA beads and incubate on a rocker for 2 h at 4°C.
  e. Wash SA-sample beads with 500 μL of Pulldown Wash for 5 min at 4°C.
  f. Use a magnetic rack to pull the beads to the bottom of the tube and extract the Pulldown Wash. Repeat the wash step 2 more times.
  g. Wash SA-sample beads with 500 μL of pre-chilled PBS for 5 min at 4°C.
  h. Use a magnetic rack to pull the beads to the bottom of the tube and extract the Pulldown Wash. Repeat the wash step 2 more times.
  i. Leave about 50 μL of PBS in the tube with SA-sample beads, and store it at −80°C.

***Note:*** For the quality control of samples, one SDS-PAGE gel with silver staining is recommended for each run of preparation (see Figure 2 for examples).

**Pause point:** The SA-sample beads mix can be stored at −80°C while the quality is being checked. Do not freeze-thaw multiple times.

**Figure 2:**
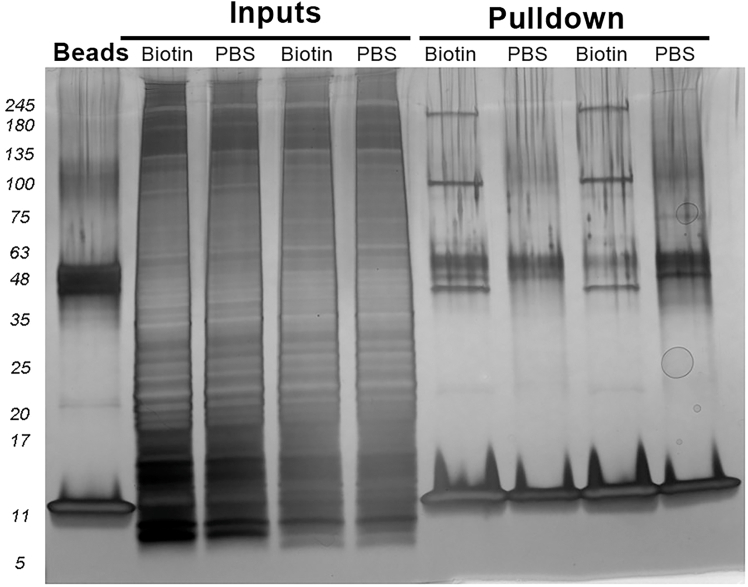
Quality validation of biotinylated protein pulldown Whole cell lysates and pulldown samples were separated by SDS-PAGE and visualized by silver staining. The eluate from streptavidin (SA) beads is shown as a negative control.

### On-beads digestion

**Timing: 3 days**

Digestion of proteins bound to the streptavidin beads after pull down. This protocol was modified from a published protocol.^3^ A silver-stained gel with molecular weight ladder is used to estimate the protein amount bound to the beads (see Figure 2).

5. Pretreat proteins on the SA beads.
  a. Use a magnetic rack to pull the beads to the bottom of the tube.
  b. Carefully remove the PBS from the sample.
    ***Note:*** Take care not to transfer beads during PBS removal. Store the removed PBS in a new tube in case troubleshooting is needed. Store PBS supernatant at −20°C until mass spectrometry data is processed.
  c. Resuspend the beads with 150 μL digestion buffer (50 mM ammonium bicarbonate, Sigma 09830).
    ***Note:*** For a pulldown using 50 μL of magnetic SA beads, 150 μl of digestion buffer will be used. If more beads are used, then the digestion buffer to beads ratio should be adjusted so it is 3 to 1.
  d. Use pH paper to ensure the resuspended beads sample is approximately pH 8.
    **CRITICAL:** Proper pH value is important for effective digestion. Adjust pH if needed.
6. Reduction and Alkylation.
  a. Add 1 mM dithiothreitol (DTT, Sigma D5545) to the resuspended beads sample, vortex, and incubate on a rocker at 20–22°C for 30 min.
  b. Centrifuge in a benchtop centrifuge for 5–10 sec to ensure all beads are in the resuspension medium.
  c. Add 5 mM iodoacetamide (IAA, Sigma I1149) to the DTT/beads mixture, vortex, and incubate on a rocker at 20–22°C for 30 min in the dark.
  d. Centrifuge in a benchtop centrifuge for 5–10 sec to ensure all beads are in the resuspension mixture.
7. Digestion of proteins on the SA beads.
  a. Digest beads with 2 μg of Lysyl Endopeptidase (Wako, 121–05063) for 16–20 h on a rocker at 20–22°C.
  b. Centrifuge in a benchtop centrifuge for 5–10 sec the following day.
  c. Add 2 μg of trypsin (Thermo Scientific 90059) to the beads mixture and allow to digest for 16–20 h on a rocker at 20–22°C.
  d. Desalt the resulting peptides with HLB column (Waters WAT094225) the next day.
  e. Vacuum dry the desalted peptide samples.

**Pause point:** The dried peptide samples can be stored at −20°C. Reserve an aliquot of a single digested sample for a pilot mass spectrometry run before processing all samples. If the silver-stained gel confirms successful pull down but peptide detection is low, consider the on-beads digestion step as a potential source of inefficiency. **Troubleshooting 6**.

### Identify biotinylated proteins by mass spectroscopy (nano-UPLC-MS/MS)

**Timing: 1 day**

Mass spectrometry was performed by the Integrated Proteomics Core Facility at Emory University. The data acquisition by nano-UPLC-MS/MS protocol was adapted from a published procedure.^4^^,^^5^ Some membrane proteins can be predominantly hydrophobic, which makes them more challenging to analyze by mass spectrometry. Because of this, it is crucial to carefully adjust both the mass spectrometer settings and the LC gradient to ensure efficient detection and separation. This protocol provides optimized conditions specifically useful for neuron derived membrane fractions, helping improve peptide recovery and identification. However, depending on the particular mass spectrometry instrument and chromatography system used, these parameters may still require additional optimization.

8. Sample loading.
  a. Resuspend derived peptides in 0.1% trifluoroacetic acid loading buffer.
    ***Note:*** Silver staining must be performed to determine the protein estimation. Peptide resuspension volume and loading amount are determined by the protein estimation on a silver stained gel.
  b. Separate peptide mixtures on a self-packed 1.7 μm CSH Waters resin column (15 cm x 100 μm internal diameter (ID); New Objective, Woburn, MA) attached to a Dionex 3000 RSLCnano system and monitor on an Orbitrap Eclipse Tribrid Mass Spectrometer (Thermo Scientific, San Jose, CA).
  c. Elute the sample over a 45 min gradient at a rate of 1.25 μL/min (MS-buffer A: 0.1% formic acid in water, MS-buffer B: 0.1% formic acid in acetonitrile) (see Figure 3).
    i. Start with 1% buffer B.
    ii. After 6 s, increase to 8%.
    iii. Gradually increase from 8% to 40% over a period of 42.5 min.
    iv. Increase to 99% within 0.5 min and maintain at 99% for another 2 min.

**Figure 3:**
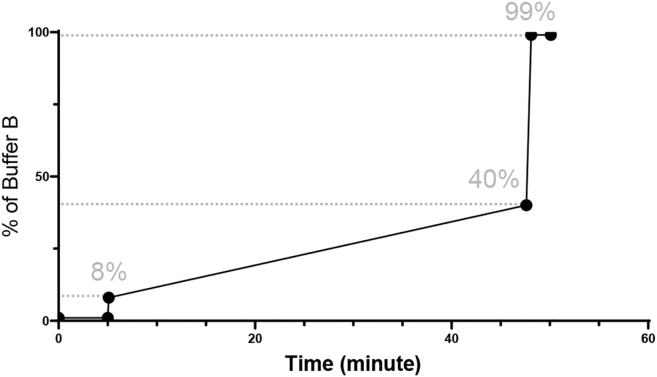
HPLC gradient settings for membrane and membrane-associated protein identification The gradient begins at 1% buffer B and increases to 8% within the first 6 seconds. From there, buffer B is gradually raised from 8% to 40% over 42.5 min. The gradient then rapidly increases to 99% within 0.5 min and is maintained at 99% for an additional 2 min.
  d. The settings of Orbitrap Fusion Lumos Mass Spectrometer.
    i. Operate the mass spectrometer in data dependent mode at top speed mode with a cycle time of 3 s.
    ii. Collect survey scans in an Orbitrap with a 60,000 resolution, 400 to 1,600 m/z range, 400,000 automatic gain control (AGC), 50 ms max injection time and rf lens at 30%.
    iii. Collect the higher energy collision dissociation (HCD) tandem mass spectra in the Orbitrap with a 15,000 resolution, collision energy of 30%, an isolation width of 1.6 m/z, AGC target of 50,000, and a max injection time of 22 ms.
    iv. Set the dynamic exclusion to 30 s with a 10 ppm mass tolerance window.
      ***Note:*** The LC-MS/MS system should be checked before running samples. The Integrated Proteomics Core Facility at Emory University routinely uses the HeLa cell digest to check their LC-MS/MS system.
  e. Manually scan all chromatograms for quality and trypsin auto-digestion peaks as control.
9. Database search.
  a. Use Proteome Discoverer 2.1 against 2020 mouse UniProtKB/Swiss-Prot database (17,041 target sequences) to search for protein spectra.
    ***Optional:*** The searching database depends on the cell types used in this process. For example, when hiPSC neurons are used, non-abundant human UniProtKB/Swiss-Prot database should be used for searching.
  b. Search the database for the following parameters: fully trypsin restriction, precursor mass tolerance (±20 ppm), and fragment mass tolerance (±0.05 Da). Methionine oxidation (+15.99492 Da), asparagine and glutamine deamidation (+0.98402 Da), and protein N-terminal acetylation (+42.03670 Da) were variable modifications (up to 3 allowed per peptide); cysteine was assigned a fixed carbamidomethyl modification (+57.021465 Da).
  c. Use Percolator to filter the peptide spectrum matches to a false discovery rate of 1%.
  d. Check the final search output for missed cleavage, which was 4% in total for 1 and 2 miscleaved tryptic sites.

## Expected outcomes

After biotinylation, the quality of the samples should be verified before proceeding with the pulldown experiment. For most cell types, the efficiency of labeling can be assessed by performing a Western blot on whole-cell lysates using fluorescently conjugated streptavidin (SA) (Figure 1A). Specifically, for neurons, immunofluorescence staining with fluorescently labeled SA provides a clear visualization of surface protein labeling on both the cell bodies and neurites (Figure 1B).

Once the pulldown is completed, silver staining of SDS–PAGE gels can be used to confirm that enough proteins have been captured by the SA beads. Because the total protein yield may be low in certain cell types, such as primary neuron cultures, silver staining is recommended for its high sensitivity. Endogenous biotinylation of proteins and aberrant binding to streptavidin beads can produce non-specific bands, hence a SA-beads-only sample should be included as a negative control (Figure 2).

**Figure 4:**
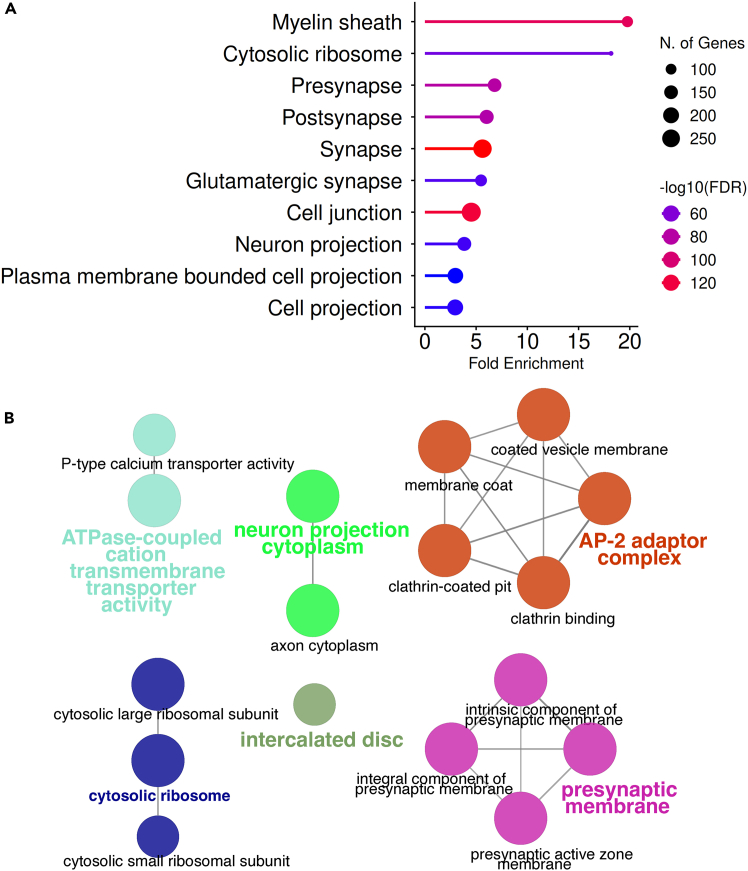
GO analysis of purified membrane fractions from neuronal cultures labeled by surface biotinylation (A) Gene set enrichment analysis indicates significant enrichment of membrane and membrane-associated proteins. (B) Gene network analysis highlights two major clusters of interest: the AP-2 adaptor complex and cytosolic ribosomal proteins.

Subcellular localization cluster analysis was performed after obtaining the mass spectrometry data, revealing that the most abundant group corresponded to synapse and neuron projection (Figure 4A). Molecular function analysis of the same dataset showed that membrane transporter proteins were significantly enriched (Figure 4B), as expected. Interestingly, components related to local translation and endocytosis were also identified (Figure 4B), suggesting that elements of the translation machinery and endocytic vesicles are positioned in close proximity to the neuronal cell membrane.

## Limitations

Compared with traditional cell membrane fractionation methods, the surface protein biotinylation and streptavidin (SA) pulldown approach involves additional steps and appears more complex. Therefore, quality control examinations, as described in the expected outcomes section, are essential.

The NHS group and the disulfide bond in Sulfo-NHS-SS-Biotin are sensitive to humidity and air. It is critical to store the Sulfo-NHS-SS-Biotin powder under anhydrous conditions and to prepare a fresh biotin solution immediately before adding it to cultured neurons. If you must prepare a stock solution of Sulfo-NHS-SS-Biotin, make a high concentration stock and aliquot it into small, single-use portions. Dilute each aliquot to the working concentration just before use, and use the reagent within a few hours. It is also critical to verify the labeling efficiency after the reaction.

Although the SA-beads pulldown is a relatively standard procedure in most laboratories, variations in handling can still affect the accuracy of quantitative analyses. To minimize variability, at least three biological replicates should be performed, and samples from the same group should be pooled for mass spectrometry (MS) analysis. For neuronal cultures, where the total protein yield is typically low, multiple rounds of preparation may be necessary to obtain sufficient material for MS analysis.

## Troubleshooting

### Problem 1

Neurons are clumpy and have abnormal morphology. Neurons are detached from the culture dish.

### Potential solution

• Poor coating can cause neurons to clump together and develop abnormal morphology, and in some cases, they may even detach from the culture dish. For high-quality cortical neuron cultures, use high molecular weight poly-L-lysine (PLL >300,000).
• To achieve optimal coating, dissolving PLL in alkaline borate buffer (pH 8.5) helps ensure full activation of the positive charges on the PLL molecules and promotes even coating of the dish surface. Borate buffer is slightly alkaline so if plates are not thoroughly washed after coating, residual borate buffer can affect the pH of the attachment medium.
• Neurons are extremely sensitive to environmental changes. Pre-incubating the PLL-coated dishes with MEM/FBS attachment medium prior to plating neurons will help equilibrate the pH of the plating medium, thus reducing neuronal shock.
• Although there are many attachment media, MEM/FBS is recommended for mouse cortical neurons.

### Problem 2

Neurons look unhealthy and are not proliferating as expected. There is an overgrowth of astrocytes and other unwanted cells.

### Potential solution

• Astrocytes typically originate from the meninges of the mouse brain. If dissections are not precise, some meningeal tissue can remain in the single-cell suspension. Perform dissections under magnification and remove as much meningeal tissue as possible during dissections to help minimize contamination.
• Astrocytes can proliferate in the culture dish and compromise the purity of neuronal cultures. If astrocytes are still visible the day after plating, certain inhibitors can be used, such as CultureOne (Thermo Scientific, Cat. A3320201). Other mitotic inhibitors, including FudR and BrdU, have also been reported to reduce glia proliferation, but they may affect neurons in some experiments. Therefore, it is best to test these reagents in your specific culture conditions before applying them routinely.

### Problem 3

Neurites are damaged and neurons detached after washing.

### Potential solution

• Rinsing with PBS can easily damage neurites or detach neurons from the culture dish. This can lead to contamination of cytosolic proteins in the membrane fraction and results in poor or no recovery of membrane proteins. Use pre-chilled PBS supplemented with calcium and magnesium ions (PBS/Ca/Mg) during the rinsing and biotinylation steps to prevent neuron detachment.
• Do not use trypsin or other enzymatic digestion methods to dissociate neurons.

### Problem 4

The protein concentration from biotinylate and lyse neuronal procedure is below 0.8 mg/mL.

### Potential solution

• Low protein yield may be caused by insufficient amount of cells plated, cell loss during culture, unhealthy neurons, or improper protein harvesting.
• Regularly check the morphology of the cultured neurons and monitor cell numbers during medium changes.
• Handle the dishes gently during biotinylation and washing steps to avoid detaching cells.
• If necessary and feasible, plate a higher number of cells at the beginning to ensure adequate protein yield.

### Problem 5

No bright SA staining signal is detected in either the Western blot or the immunofluorescence staining.

### Potential solution

• When performing *in situ* biotinylation, pH is a major factor influencing labeling efficiency. The reaction should be carried out at pH 7–9. Acidic pH protonates amine groups (–NH_3_^+^), which significantly reduces the biotinylation efficiency.
• To achieve optimal biotinylation efficiency, prepare a fresh EZ-Link Sulfo-NHS-SS-Biotin solution from powder each time. Alternatively, prepare a 100 mM stock solution of EZ-Link Sulfo-NHS-SS-Biotin in PBS containing calcium and magnesium (PBS/Ca/Mg). Aliquot the stock into small volumes sufficient for a single experiment and store at −20°C. Before using it, thaw one aliquot on ice and dilute the biotin in PBS/Ca/Mg while rinsing neurons.

### Problem 6

The on-beads digestion was not complete. Where is the biotinylated protein?

### Potential solution

• The PBS washing steps are critical for MS sample preparation following the Buffer A washes. Thorough removal of the Pulldown Wash is essential for on-bead digestion step. Detergents can cause ion suppression.
• An alternative approach is to elute proteins from the beads by incubating with DTT, which cleaves the biotin-amino acid bonds. However, if the samples are intended for other biochemical analyses, such as Western blotting, the proteins can be eluted using an equal volume of 2X sample buffer supplemented with DTT.
• Proper pH value is important for effective digestion. Adjust pH if needed.

## Resource availability

### Lead contact

Further information and requests for resources and reagents should be directed to and will be fulfilled by the lead contact, Gary J. Bassell (gary.bassell@emory.edu).

### Technical contact

Technical questions on executing this protocol should be directed to and will be answered by the technical contact, Liang Shi (liang.shi@emory.edu).

### Materials availability

• This study did not generate new unique reagents.
• Time-pregnant mice can be generated in-house using time-mating breeding technique for accurate gestational timing. Alternatively, time-pregnant mice can be ordered from vendors such as Charles River Laboratories or The Jackson Laboratory. If ordering time-pregnant mouse for neuronal collection, the mouse must arrive in the facility by E15 to allow at least 1 day of acclimation before E17 collection day.

### Data and code availability

• The mass spectrometry proteomics data generated from this study are deposited in the ProteomeXchange Consortium via the PRIDE55 partner repository with the dataset identifier PXD065502 (see key resources table).
• No code was generated for this protocol.
• Any additional information required to reanalyze the data reported in this paper is available from the lead contact upon request.

## Acknowledgments

This work was supported by the following NIH grants: the National Institute of Mental Health (NIMH) R03MH135518 (G.J.B.), the National Institute of Neurological Disorders and Stroke (NINDS) R21NS143271 (G.J.B.), the National Institute on Aging (NIA) RF1AG060285 (V.F.) and the National Institute of Environmental Health Sciences (NIEHS) R01ES034796 (V.F.). This work was supported in part by the Emory Integrated Proteomics Core (RRID: SCR_023530).

## Author contributions

L.S., V.F., and G.J.B. conceived the study, designed the experiments, and analyzed and interpreted the data. L.S. performed most of the experiments. G.N.N. assisted with drafting and formatting the manuscript. P.B. performed the MS analysis. L.S., V.F., and G.J.B. wrote the paper with input and edits from co-authors. V.F. and G.J.B. are co-senior authors.

## Declaration of interests

G.J.B. serves on the advisory board of *Cell Reports*.
